# The Multifaceted Role of Extracellular Vesicles in Glioblastoma: microRNA Nanocarriers for Disease Progression and Gene Therapy

**DOI:** 10.3390/pharmaceutics13070988

**Published:** 2021-06-29

**Authors:** Natalia Simionescu, Radu Zonda, Anca Roxana Petrovici, Adriana Georgescu

**Affiliations:** 1 Center of Advanced Research in Bionanoconjugates and Biopolymers, “Petru Poni” Institute of Macromolecular Chemistry, 41A Grigore Ghica Voda Alley, 700487 Iasi, Romania; natalia.simionescu@icmpp.ro (N.S.); zonda.radu@icmpp.ro (R.Z.); petrovici.anca@icmpp.ro (A.R.P.); 2 “Prof. Dr. Nicolae Oblu” Emergency Clinical Hospital, 2 Ateneului Street, 700309 Iasi, Romania; 3 Department of Pathophysiology and Pharmacology, Institute of Cellular Biology and Pathology “Nicolae Simionescu” of the Romanian Academy, 8 B.P. Hasdeu Street, 050568 Bucharest, Romania

**Keywords:** extracellular vesicles, glioblastoma, microRNA, biomarkers, nanocarriers, therapy

## Abstract

Glioblastoma (GB) is the most aggressive form of brain cancer in adults, characterized by poor survival rates and lack of effective therapies. MicroRNAs (miRNAs) are small, non-coding RNAs that regulate gene expression post-transcriptionally through specific pairing with target messenger RNAs (mRNAs). Extracellular vesicles (EVs), a heterogeneous group of cell-derived vesicles, transport miRNAs, mRNAs and intracellular proteins, and have been shown to promote horizontal malignancy into adjacent tissue, as well as resistance to conventional therapies. Furthermore, GB-derived EVs have distinct miRNA contents and are able to penetrate the blood–brain barrier. Numerous studies have attempted to identify EV-associated miRNA biomarkers in serum/plasma and cerebrospinal fluid, but their collective findings fail to identify reliable biomarkers that can be applied in clinical settings. However, EVs carrying specific miRNAs or miRNA inhibitors have great potential as therapeutic nanotools in GB, and several studies have investigated this possibility on in vitro and in vivo models. In this review, we discuss the role of EVs and their miRNA content in GB progression and resistance to therapy, with emphasis on their potential as diagnostic, prognostic and disease monitoring biomarkers and as nanocarriers for gene therapy.

## 1. Introduction

Glioblastoma (GB) is the most aggressive form of brain cancer in adults, characterized by fast growth and invasiveness, high tumor heterogeneity, poor survival and lack of effective therapies [1,2,3]. The diagnosis and classification of brain tumors have undergone several modifications over the last two decades. Thus, the latest classification of central nervous system (CNS) tumors released by the World Health Organization (WHO) [4] takes into account molecular markers along with histological assessment and clinical presentation into the diagnosis and classification of GB. In this regard, it has been demonstrated that GBs with identical histopathological classification, but of a different molecular subtype, have distinctive clinical outcomes and treatment responses: the Proneural subtype is associated with longer survival and low treatment response compared to other subtypes, while Classical and Mesenchymal subtypes respond significantly better to aggressive treatment [5,6]. Following diagnosis, the current standard of treatment for GB includes maximum safe surgical resection (often aided by 5-aminolevulinic acid (5-ALA)-induced tumor fluorescence), radiotherapy and chemotherapy using temozolomide (TMZ) or other agents [7,8,9,10,11].

Despite recent therapeutic advances and improved imaging techniques, *de novo* GB diagnosis is frequently done in advanced stages of the disease, when the impact on patients’ quality of life is severe. Furthermore, recurring GB tumors are still difficult to manage, and magnetic resonance imaging (MRI) follow-ups are expensive and sometimes misleading, as it is difficult to distinguish between recurrence and pseudo progression. Despite ongoing efforts to develop new diagnostic and therapeutic tools, minimal advances have been made, and no reliable biomarkers are being used in clinical practice [12]. Therefore, there is a need for minimally invasive, easy to measure and cost-effective biomarkers for early diagnosis of GB and therapeutic response monitoring. The advancements in molecular biology in the last decades have led to the discovery of new potential biomarkers, among which microRNAs (miRNAs) seem to be the most promising ones.

MiRNAs are small, single-stranded, non-coding RNAs that regulate gene expression post-transcriptionally by inhibiting translation and/or promoting messenger RNA (mRNA) degradation through specific pairing with target mRNAs [13]. MiRNAs are stress response molecules, have modified expression levels during disease progression and are known to be involved in the initiation and development of various types of cancer [14,15]. Furthermore, miRNAs have been shown to circulate in the blood stream and cerebrospinal fluid (CSF), associated with extracellular vesicles (EVs), lipoproteins or protein complexes, and their circulating profiles reflect their modified tissue expression or an increased intercellular communication [16,17]. This, combined with the fact that miRNAs are relatively easy to measure in biological fluids, supports their potential use as biomarkers for diagnosis, prognosis and therapeutic response monitoring of CNS malignancies. However, many studies have attempted to identify specific serum or CSF miRNAs as biomarkers for brain tumors, including GB [18], but their collective findings fail to identify reliable biomarkers that can be applied in clinical settings. Serum biomarkers are easy to measure and can be useful in clinical practice, but EVs have a more disease- and tissue-specific cargo and could differentiate between pathologies more accurately.

EVs represent a heterogenous group of lipid vesicles that are secreted by numerous cell types, under physiological or pathological conditions, exhibit specific markers and transport particular molecules from their cells of origin, including miRNAs [19,20,21,22,23,24]. Furthermore, EVs bind and fuse with their target cells, delivering their cargo and promoting horizontal malignancy into adjacent tissues [23,25], as well as resistance to therapeutic interventions [26,27,28,29,30,31]. On the other hand, EVs derived from healthy cells have been shown to improve pathological conditions in recipient cells [32,33]. Considering the ability of miRNAs to target multiple transcripts, EV-mediated transfer of miRNAs to recipient cells could have an extensive impact.

EVs can be isolated from biological fluids [34,35,36,37,38,39,40,41] or cell culture medium [42], providing an extensive platform for studying pathological processes. Moreover, EVs have been shown to contain a significantly distinct miRNA signature compared to their cells of origin, suggesting a selective miRNA packaging into EVs [24], and their number and miRNA content change under pathological conditions [23,43,44]. These aspects could be exploited in a clinical setting as EVs have been shown to have diagnostic potential in various pathologies, including GB [26,45], as well as biomarker potential for treatment response monitoring and disease recurrence [46,47,48,49,50,51].

Due to the ability of EVs to cross the blood–brain barrier [52,53] and to transfer their cargo to a wide array of cells [23,25,32,33], they could be used as therapeutic tools in GB. This possibility opens up many new avenues in cancer treatment, aided by the fact that EVs can be enriched in endogenous [54] or synthetic miRNAs [55], or miRNA inhibitors [56,57]. The production and clinical use of EV-based therapeutics depend on numerous safety, biological and manufacturing aspects and are still not clearly regulated [58]. Despite current limitations and drawbacks, EV-based miRNA nanocarriers could represent an important adjuvant in GB therapy, combined with the current standard of treatment.

In this review, we highlight the main aspects of GB pathology, including cellular and molecular insights into disease progression, the properties and role of EVs in GB progression and the potential of EV-associated miRNAs as biomarkers and therapeutic tools in GB.

## 2. Pathogenesis of Glioblastoma

Before 2016, the diagnosis and classification of brain tumors was done almost exclusively on the basis of histological evidence, regardless of clinical manifestations [59]. In the case of GB, the 2007 World Health Organization (WHO) Classification of Tumors of the Central Nervous System (CNS) defines it as an astrocytic tumor and a grade IV neoplasm, which designates “cytologically malignant, mitotically active, necrosis-prone neoplasms typically associated with rapid pre- and postoperative disease evolution and a fatal outcome” [59]. Following the 2014 meeting of the International Society of Neuropathology held in Haarlem, Netherlands, a revised fourth edition of the WHO Classification of Tumors of the CNS was released in 2016 [4]. This update integrates molecular data into the diagnostics and classification of brain tumors, defining GBs as “diffuse astrocytic and oligodendroglial tumors” and establishing a new distinction between GB subtypes based on isocitrate dehydrogenase (IDH) mutations: IDH-wildtype (giant cell GB, gliosarcoma and epithelioid GB), IDH-mutant and NOS (not otherwise specified) [4]. This distinction acknowledges that GBs with identical histopathological classification, but of a different molecular subtype, may have distinct clinical outcomes and treatment responses, and that their molecular characteristics could partially explain disease progression [60,61]. Moreover, the recently created Consortium to Inform Molecular and Practical Approaches to CNS Tumor Taxonomy (cIMPACT-NOW) has published seven papers which outline the proposed modifications in the upcoming fifth edition of the WHO Classification of Tumors of the CNS [62]. According to this update, IDH-mutant astrocytoma (WHO grade IV), with CDKN2A/B locus homozygous deletion as molecular marker, previously classified as IDH-mutant GB [4,11,63], is now classified as “astrocytoma, IDH-mutant, WHO grade 4 and no longer as glioblastoma” [62,64].

In the 2016 classification, IDH-wildtype GBs are considered primary GBs, which represent more than 90% of GBs, localize most frequently in cerebral hemispheres, are characterized by extensive necrosis and manifest clinically *de novo*, usually in older patients [2,4,8]. On the other hand, IDH-mutant GBs are considered secondary GBs, which evolve from lower grade precursors and have longer median survival rates than IDH-wildtype GBs [4]. Giant cell GBs represent 1–5% of all GBs, occur in younger patients and are comprised of multinucleated giant cells [65]. Gliosarcomas represent 2% of all GBs and exhibit high rates of extracranial metastases, while epithelioid GBs occur mainly in children and young adults and are comprised of large epithelioid cells with abundant cytoplasm and vesicular chromatin [65]. According to the latest European Association of Neuro-Oncology guidelines, “glioblastoma is now defined as a diffuse astrocytic glioma with no mutations in IDH genes nor histone H3 genes and is characterized by microvascular proliferation, necrosis and/or specific molecular features, including *TERT* promoter mutation, *EGFR* gene amplification and/or a +7/−10 cytogenetic signature” [11].

Given the variations in GB nomenclature in the literature, in this review we will use the nomenclature established by the 2016 WHO Classification of Tumors of the CNS, as this is the most common.

Overall, GB is the most aggressive form of brain cancer in adults, characterized by fast growth and invasiveness, extensive vascularization and hypoxic niches rich in cancer stem-like cells, as well as remarkably high tumor heterogeneity and poor response to conventional therapies [1,2,3]. GB symptoms depend on the tumor location and size and often include headaches, seizures, focal deficits and neurocognitive impairment [11]. Following diagnosis based on clinical presentation and imaging techniques (computed tomography (CT), MRI), the current standard of treatment for GB includes maximum safe surgical resection (often aided by 5-ALA-induced tumor fluorescence), radiotherapy and chemotherapy using TMZ or other agents [7,8,9,10,11].

### 2.1. Epidemiology and Etiology of Glioblastoma

Globally, brain tumors are the nineteenth most common neoplasms (1.7% of all new cancer cases with a median yearly incidence of 3.9/100,000 persons), having the highest incidence in Northern Europe (in particular Lithuania and Norway), followed by Australia, United States and Canada [66]. GB patients have a median survival expectancy of approximately 12 months and suffer from extensive cognitive and emotional deficits [11]. GB survival has been inversely correlated with age at diagnosis, 5 year survival decreasing from 5% (all patients) to 2% in patients over 65 years old [67]. Furthermore, GB survival after standard treatment appears to depend on gender, as the 5-year survival rate in females is higher compared to male patients [68,69]. Additionally, pre-operative Karnofsky Performance Status (KPS) scores of GB patients were found to be directly correlated with overall survival [70]. Regarding ethnicity, it has been shown that Caucasians have the highest incidence and lowest survival rates, followed by Hispanic, Asian and African patients, respectively [71,72].

To date, the only confirmed exogenous risk factor for GB is the exposure of the head and neck to ionizing radiation (therapeutic [73] or otherwise). However, some studies have shown associations of glioma occurrence with hereditary syndromes (Li-Fraumeni syndrome, Neurofibromatosis Type 1, Turcot’s Syndrome, familial history of glioma), gender (males more affected than females), increased age, ethnicity (Caucasians more affected than Africans or Asians), taller adult height, epilepsy, seizures or convulsions [66,74,75,76,77,78,79]. In contrast, history of allergies, autoimmune diseases and viral infections (e.g., colds, flu, herpes virus or varicella zoster virus) have been shown to be inversely correlated with glioma risk, suggesting the involvement of immunological factors in glioma development [80,81].

Some studies have associated higher glioma risk with various occupations such as physicians, firefighters, farmers, anatomists, pathologists, embalmers, janitors, motor vehicle operators, painters, food processors, social service workers, teachers, metal processing and shaping workers, construction workers, etc., but these results have not been confirmed in other populations [77,82]. Furthermore, the occupations themselves are seemingly random, and their odds ratios and confidence intervals are relatively low [82], suggesting a cautious approach to their interpretation. Despite some results reported in the literature, occupational exposure to extremely low frequency magnetic fields is not considered as a GB risk factor by the International Agency for Research on Cancer (IARC) [79]. Moreover, the INTERPHONE international case–control study [83], coordinated by IARC, did not report any significant association between glioma risk and mobile phone use.

Some studies showed that specific chemicals such as asbestos, benzene, mineral or lubricating oil, plastics, rubber products, arsenic, mercury, petroleum products and pesticides were associated with high glioma risk, but the correlation is weak at best [77,82]. In contrast, Carréon et al. [84] reported no significant association between exposure to 12 widely used pesticides and glioma risk in women. Additionally, there was no causal link found between smoking and glioma development [85].

Regarding dietary habits, increased glioma risk was associated with frequent intake of products with high levels of nitrite, such as cured meat, while diets rich in fresh fruits and vegetables were inversely correlated with glioma risk, but the results are still inconclusive [78,79]. On the other hand, decreased GB risk was found to be associated with regular aspirin use [79].

Overall, some of the risk factors associated with GB may reflect socioeconomic differences rather than actual correlation [77]. Interestingly, Porter et al. [86] reported a strong correlation between higher socioeconomic status and GB risk.

### 2.2. Cellular and Molecular Insights into Glioblastoma Development

GBs are extremely heterogeneous tumors, both macroscopically and microscopically [87]. On CT or MRI scans, GBs appear as irregular lesions, usually presenting central necrosis and perilesional edema, and angiography reveals extremely abnormal vasculature [88]. The histopathological hallmarks of GB include necrosis, calcifications and microvascular proliferation [87,89,90,91]. In addition, histological analysis of GB revealed pleomorphic cells, hyperplasia of endothelial cells and pericytes, lymphocytic infiltration, hemorrhages, thrombosis, angiocentric structures, desmoplasia, anaplasia, high mitotic rates, etc. [74,87,90,91]. Usually, GBs consist of glial-like cells or cells with astrocytic features, but some tumors exhibit intratumoral heterogeneity and present an oligodendroglial–astrocytic phenotype [2].

GB tumors appear to originate from neural stem cells [2,92] and contain various types of tumor and stromal cells, including glioma stem-like cells (GSCs), microglia, astrocytes, immune cells, fibroblasts, endothelial cells and pericytes, which create a microenvironment that facilitates tumor progression and resistance to therapy [93,94,95]. GSCs represent a population of cells with stem cell-like properties, characterized by self-renewal and the capacity to initiate tumors after transplantation [61,74,94,96,97]. Furthermore, GSCs have been shown to contribute to GB’s cellular heterogeneity and are involved in therapeutic resistance by conferring radio- and chemoresistance, stimulating angiogenesis and invasion and inducing recurrence [61,74,94,96,97]. GSCs exhibit several markers, such as CD133, CD15, CD44, integrin alpha 6 (ITGA6), L1 cell adhesion molecule (L1CAM) and SRY-Box Transcription Factor 2 (SOX2), but no specific set of markers has been identified [61,74]. Microglia cells represent an important cell population in GB tumors and have been shown to stimulate invasion through secretion of matrix metalloproteases (MMP2 and MMP9), which determine the degradation of extracellular matrix components, and transforming growth factor β (TGF-β) and other cytokines, which activate specific signaling pathways [94,95]. Astrocytes are also abundant in GB microenvironment, but their role in tumor development and progression is still unclear [94]. However, it has been determined that astrocytes become activated by GB cells and modulate the tumor microenvironment by secreting soluble factors, degrading extracellular matrix components and promoting tumor invasion [95]. Tumor-associated macrophages have been shown to promote tumor growth, while endothelial cells promote GSC proliferation and migration through paracrine signals [95].

The extracellular matrix also plays an important role in GB progression by promoting adhesion, proliferation and metastasis of tumor cells through fibronectin, laminin and hyaluronic acid, as well as modulating specific signaling pathways [95]. In addition, GB cells secrete soluble factors, such as chemokines, interleukins, growth factors, proteases and miRNAs, and release EVs, which affect surrounding cells, inducing their active participation in tumor growth [93,95,98,99]. These cellular and molecular components constitute the tumor microenvironment, which has a crucial role in GB progression and determines disease recurrence even after the most aggressive treatment [100].

**Figure 1:**
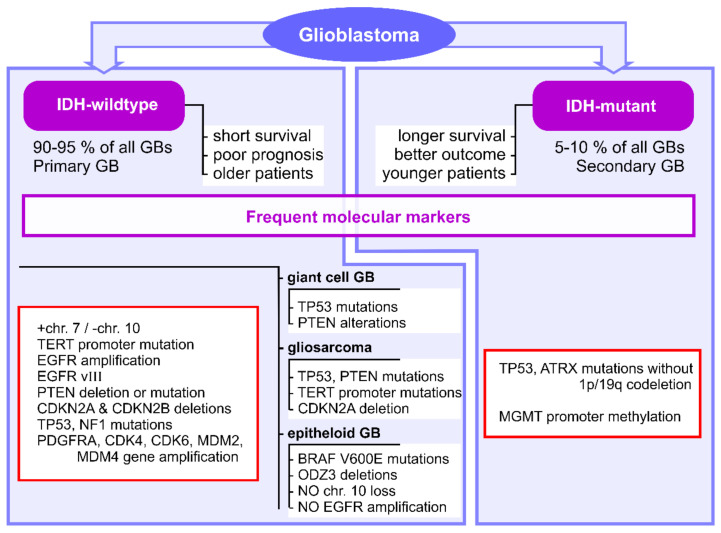
Frequent molecular markers identified for GB clinical subtypes. IDH1, isocitrate dehydrogenase 1; GB, glioblastoma; TERT, telomerase reverse transcriptase; EGFR, epidermal growth factor receptor; PTEN, phosphatase and tensin homolog; CDKN2A, cyclin-dependent kinase inhibitor 2A; CDKN2B, cyclin-dependent kinase inhibitor 2B; NF1, neurofibromin 1; PDGFRA, platelet-derived growth factor receptor A; CDK4, cyclin-dependent kinase 4; CDK6, cyclin-dependent kinase 6; MDM2, mouse double minute 2 homolog; MDM4, mouse double minute 4 homolog; MGMT, O6-alkylguanine DNA alkyltransferase.

Several molecular markers have been identified for GB subtypes and have been included in their diagnosis and management guides: IDH mutation, O6-alkylguanine DNA alkyltransferase (MGMT) promoter methylation, chromosome 1p/19q codeletion, TP53 mutation, phosphatase and tensin homolog (PTEN) mutation, cyclin-dependent kinase inhibitor 2A (CDKN2A) deletion, platelet-derived growth factor receptor A (PDGFRA) amplification, neurofibromin 1 (NF1) mutation, mouse double minute 2 homolog (MDM2) amplification, +7/−10 genotype, telomerase reverse transcriptase (TERT) promoter mutation and epidermal growth factor receptor (EGFR) amplification [11]. The main marker, which is currently used in clinical practice, is represented by the IDH genes mutation status, which discriminates between IDH-mutant and IDH-wildtype GB in terms of diagnostic and disease progression [4,11]. Patients with IDH-mutant GB (approximately 5–10% of all GBs) have longer median survival rates and better outcomes than patients with IDH-wildtype GB, which are usually older and have poor prognosis [74,92,101] (Figure 1).

The DNA repair protein MGMT is responsible for TMZ resistance in GB, and the methylation of its promoter has been linked to better outcomes for patients [74,87]. In addition, CDKN2A and CDKN2B deletions indicate poor prognosis in IDH-mutant astrocytoma [11]. The oncogenes EGFR and PDGFRA are often overexpressed in primary GBs and stimulate tumor proliferation [92].

IDH-wildtype GBs commonly exhibit concomitant gain of chromosome 7 and loss of chromosome 10 (+7/−10 genotype), TERT promoter mutation and EGFR gene amplification [11] (Figure 1). The presence of +7/−10 genotype and TERT promoter mutation have been shown to be indicative of poor prognosis in GB [92,102]. In addition, these tumors often present PTEN deletion or mutation and CDKN2A and CDKN2B deletions, as well as TP53 and NF1 mutations, PDGFRA, CDK4, CDK6, MDM2 and MDM4 gene amplifications [63] (Figure 1). Almost half of IDH-wildtype GBs exhibit deletion of EGFR exons 2-7, resulting in the expression of EGFR variant III (EGFRvIII) [63] (Figure 1). IDH-wildtype GBs include giant cell GB, gliosarcoma and epithelioid GB [4]. Giant cell GBs commonly exhibit TP53 mutations, and some tumors may present with PTEN alterations [65] (Figure 1). Gliosarcomas are characterized by TP53, PTEN and TERT promoter mutations and CDKN2A deletion, but no definitive markers have been identified [65] (Figure 1). Epithelioid GBs are uniquely characterized by BRAF V600E mutations and frequent ODZ3 deletions, but do not exhibit typical primary GB markers, such as chromosome 10 loss and EGFR amplification [4,65] (Figure 1). On the other hand, IDH-mutant GBs are characterized by TP53 and ATRX mutations without 1p/19q codeletion, as well as MGMT promoter methylation [63,102] (Figure 1).

There have been several attempts at classifying GBs into molecular subtypes in order to better understand disease progression, prognosis and therapeutic response. The Cancer Genome Atlas (TCGA), a large-scale genomic and epigenomic study, provided the possibility of analyzing a large number of samples and identifying molecular markers specific to GB subtypes. However, the analysis of these samples by various groups has yielded different GB subtype classifications.

The TCGA pilot study identified the most commonly mutated genes in GB: TP53, PTEN, NF1, EGFR, retinoblastoma protein (RB1), phosphoinositide-3-kinase regulatory subunit 1 (PIK3R1), and PIK3CA [103]. A subsequent study done by Brennan et al. [6] identified other significantly mutated genes in GB: IDH1, PDGFRA, leucine-zipper-like transcriptional regulator 1 (LZTR1), spectrin alpha 1 (SPTA1), ATRX, gamma-aminobutyric acid type A receptor subunit alpha 6 (GABRA6) and KEL, as well as amplifications in chromosome 4 (PDGFRA), chromosome 7 (EGFR, MET, CDK6) and chromosome 12 (CDK4, MDM2). Additionally, a TCGA-based study showed that GB tumors often exhibit alterations of the TP53, RB and RTK/Ras/PI3K signaling pathways [6], contributing to cancer cell migration, invasion, proliferation, differentiation and survival [104].

The analysis of large GB patient cohorts has led to partially overlapping classifications, the most commonly cited being the classification by Phillips et al. [105] and the classification by Verhaak et al. [5]. Phillips et al. [105] analyzed WHO grade III and IV astrocytoma (anaplastic astrocytoma and GB) samples and identified three molecular subtypes: Proneural, Proliferative and Mesenchymal, which were further validated on an independent set of WHO grade IV astrocytomas with necrosis. On the other hand, Verhaak et al. [5] analyzed only GB samples and classified them into four molecular subtypes: Proneural, Neural, Mesenchymal and Classical, which were further validated in an independent data set and are currently used as the main subtypes in TCGA classification. The characteristics of each subtype are summarized in Table 1.

Phillips et al. [105] determined the gene signatures specific to each subtype and showed that the three identified subtypes have significant prognostic value. This study showed that Proneural tumors were associated with longer survival than the other subtypes [105]. The Proliferative tumors overexpress markers of proliferation, while Mesenchymal tumors overexpress vascular endothelial growth factor (VEGF), VEGF receptors and platelet endothelial cell adhesion molecule (PECAM) [105]. On the other hand, Verhaak et al. [5] showed that GBs with identical histopathological classification, but of a different molecular subtype, have distinctive clinical outcomes and treatment responses: the Proneural subtype is associated with longer survival and low treatment response compared to other subtypes, while Classical and Mesenchymal subtypes respond significantly better to aggressive treatment. Briefly, the Proneural subtype is characterized by modified PDGFRA expression and IDH1 point mutations, the Neural subtype by the expression of neuron markers (neurofilament light—NEFL, GABRA1, synaptotagmin-1—SYT1 and solute carrier family 12 member 5—SLC12A5), the Mesenchymal subtype by low expression or loss of NF1, and the Classical subtype by increased amplification of EGFR and absence of TP53 mutations [5].

## 3. Extracellular Vesicles as Nano Mediators in Glioblastoma Progression

### 3.1. Biogenesis, Classification and Functional Cargo of Extracellular Vesicles

The International Society for Extracellular Vesicles (ISEV) recommends the use of “extracellular vesicles (EVs)” as “the generic term for particles naturally released from the cells that are delimited by a lipid bilayer and cannot replicate, i.e., do not contain a functional nucleus” [106]. Therefore, EVs represent a heterogenous group of vesicles that are secreted by numerous cell types, under physiological or pathological conditions [21], and can be isolated from biological fluids (such as blood, urine, breast milk, amniotic fluid, cerebrospinal fluid, semen, saliva and bronchial lavage fluid) [34,35,36,37,38,39,40,41] or cell culture medium [42].

EVs were first described in 1967 by P. Wolf [107] as “platelet-dust”, rich in phospholipids and having coagulant properties. In 1981, the term “exosome” was coined by Trams et al. [108] and EVs were characterized as “vesicles with 5′-nucleotidase activity” and “an average diameter of 500 to 1000 nm”, which are released in vitro from normal and neoplastic cells. Due to the great variability of EVs isolated by different methods from various samples, ISEV guidelines do not offer a clear classification of EVs, but instead recommend the thorough characterization of isolated EVs (physical characteristics, biochemical composition, isolation condition description, cells of origin) [106]. However, the most common classification in the literature distinguishes three main EV subtypes: exosomes, microvesicles (MVs, also called microparticles or ectosomes) and apoptotic bodies, based on their mode of biogenesis and release from cells [109]. These subtypes differ in origin and release mechanisms, size, composition and function [19]. The main characteristics of EVs are summarized in Table 2.

Exosomes are small EVs of 30–100 nm, which are formed through the endosomal pathway [21]. The inward budding of endosomes leads to the formation of intraluminal vesicles (ILVs) organized in multivesicular bodies (MVBs), followed by the fusing of MVBs with the plasma membrane and the consequent release of their contents (i.e., exosomes) into the extracellular space [21,112,116]. The exosome biogenesis pathway is not completely elucidated, but it is known to be regulated by a wide array of proteins, such as the Endosomal Sorting Complex Required for Transport (ESCRT) machinery (ESCRT-0, ESCRT-I, ESCRT-II, and ESCRT-III), Rab guanosine triphosphatases, apoptosis-linked gene 2-interacting protein X (ALIX), vacuolar protein sorting-associated protein (VPS4), neutral sphingomyelinases, phospholipase D2 (PLD2) and ADP ribosylation factor 6 (ARF6) [21,112,118]. Exosomes exhibit lipid membrane composition similar to that of the donor cell’s plasma membrane and are typically enriched in tetraspanins and heat shock proteins [115] and exhibit specific surface markers with roles in cellular adhesion and internalization, such as integrins, intercellular adhesion molecule-1 (ICAM-1) or L1CAM [119].

In contrast to exosomes, MVs represent a heterogenous population of phosphatidylserine (PS)-positive EVs (100–1000 nm) generated by the direct outward budding or blebbing of the plasma membrane of activated or apoptotic cells [21,32,115,116]. They have variable shapes and are commonly shed by platelets, endothelial cells and white blood cells [20,115]. MVs biogenesis involves actin cytoskeleton modifications (ARF6-dependent) and the asymmetrical rearrangement of phospholipids in the plasma membrane (induced by Ca^2+^-dependent enzymes), leading to the translocation of PS to the outer membrane [21]. Increased calcium concentrations, both intracellular and extracellular, improve MV formation and release, together with other factors such as hypoxia (most often present in solid tumors) and actin deamination [21]. MVs reflect the plasma membrane composition and markers of their respective origin cell, including specific surface antigens and receptors, such as CD41, CD18, EGFR, its mutant variant (EGFRvIII) or epithelial cell adhesion molecule (EpCAM) [22,120,121,122].

Apoptotic bodies represent a particular type of EVs, which are formed through cellular blebbing and fragmentation during apoptosis [21,115,116]. They present in heterogenous sizes ranging from 1 to 5 µm and contain cellular organelles, chromatin, proteins, RNA, membranes and other cytosolic contents [21,115,116].

During their biogenesis, EVs enclose specific molecules from their cells of origin, including enzymes, proteins, transcription factors, lipids and nucleic acids (DNA, mRNA, miRNA, long non-coding RNA, circular RNA, etc.) [19,20,21,22,23,24,93,112,120]. This specific packaging in EVs depends on environmental conditions, epigenetic changes, their biogenesis and other factors, and their cargo reflects the pathophysiological state of the cell of origin [19,21,23,24,32,44,123].

In the last decades, EVs have been studied extensively, revealing a complex array of functions, depending on their cells of origin and their characteristics (more specifically their cargo). Exosomes and MVs have been proposed numerous times as biomarkers for a variety of pathologies due to their specific cargo, availability in body fluids and intercellular transfer capabilities [119,124,125]. In contrast, apoptotic bodies’ formation takes place during apoptosis, leading to PS enrichment in their outer membrane which binds to Annexin V and facilitates phagocytosis [126], therefore they should not be considered as significant intercellular communication vesicles.

The phospholipid bilayer of EVs protects their cargo, providing stability, long half-life and resistance to degradation [127], while their small size offers biocompatibility and the ability to evade the immune system [112]. Furthermore, it has been shown that EVs are mediators of intercellular communication in both physiological and pathological conditions [21,93] and are even capable of crossing the blood–brain barrier [52,53]. Their functional cargo and specific characteristics offer EVs the ability to regulate numerous processes in cancer development and progression, such as cell proliferation and migration, inflammation, angiogenesis, immune suppression, invasion and metastasis [21,112]. EVs generated by tumor cells can transfer their functional cargo into recipient cells, which alters their physiological mechanisms and affects their phenotype [23,25,93,112]. In this regard, EVs of cancer cell-origin have been shown to function as protumor or antitumor agents, depending on the type of recipient cells and their response to the EV cargo [112]. Additionally, EVs have been shown to promote resistance to therapeutic interventions [26,43,128] and to have diagnostic and therapeutic potential in various pathologies, including GB [26,32,45].

### 3.2. Extracellular Vesicles and Their Associated microRNAs as Protagonists in Glioblastoma Progression

EVs represent an integral part in the physiology of different tissues and organs, including the CNS, with roles in neurodevelopment, neuroprotection, differentiation and signaling [129]. In pathological states, activated or malignant cells release EVs which maintain and exacerbate disease-related processes, such as proliferation, migration and invasion of cancer cells, angiogenesis, metastasis, resistance to apoptosis, immune escape, inflammation, etc. [112]. GB cells release EVs, which carry oncogenic factors and act as mediators in intercellular communication, leading to the formation of a pro-tumorigenic environment [129,130]. It is clear that EVs promote horizontal malignancy in GB, by transferring their cargo to neighboring cells, inducing tumor-supportive phenotypes, promoting angiogenesis and immunosuppression, increasing proliferation, modulating metabolic activity and conferring drug resistance [131,132]. GB-derived EVs promote angiogenesis by altering the functionality of endothelial cells through pro-angiogenic factors such as VEGF, fibroblast growth factor (FGF), PDGF, angiogenin, interleukins, TGF-β, other cytokines, proteases and miRNAs [131]. Furthermore, hypoxic GB cells secrete EVs containing proangiogenic cargo, which in turn influence endothelial cells to secrete soluble factors to stimulate pericytes’ and smooth muscle cells’ proliferation and GB cells’ migration [133]. Al-Nedawi et al. [22] showed that GB EVs transport EGFRvIII, the oncogenic mutant variant of EGFR frequently detected in GB tumors, which stimulates VEGF production and promotes angiogenesis. Liu et al. [134] established that MVs isolated from plasma and CSF of GB patients induce endothelial cell proliferation in vitro through activation of the Akt/beta-catenin pathway. Furthermore, GB EVs decrease the brain vascular permeability and increase brain vascular leakage by expressing Semaphorin3A [135]. GB-derived EVs also induce immunosuppressive phenotypes in tumor-associated macrophages and inhibit lymphocyte activity through various mechanisms, thus promoting tumor development [136,137,138,139]. Additionally, GB EVs induce tumor cell migration, invasion and proliferation by transferring L1CAM, annexin A2 [140,141] and chloride intracellular channel-1 (CLIC1) [142] to neighboring cells.

EVs also mediate therapeutic resistance in GB by various mechanisms. For example, Shao et al. [143] demonstrated that TMZ-resistant cells transfer their ability to chemosensitive cells through EVs carrying MGMT and alkylpurine-DNA-N-glycosylase (APNG) mRNAs, both coding key DNA damage repair enzymes. Additionally, Yu et al. [144] showed that GB cells can acquire TMZ resistance through EVs released by reactive neighboring astrocytes which carry MGMT mRNA. Furthermore, GB-derived EV transfer drug efflux pumps, such as permeability glycoprotein (P-GP) and multidrug resistance associated protein (MRP1) [145], and induce mesenchymal transition through the modulation of nuclear factor-κB/signal transducer and activator of transcription 3 (NF-κB/STAT3) signaling [146]. Moreover, it has been shown that tumors release EVs enriched in bevacizumab, an anti-VEGF antibody used in GB chemotherapy, as a clearance mechanism in therapeutic resistance [147]. Ionizing radiation stimulates EV release from GB cells, promoting a migratory phenotype in recipient cancer cells through the transfer of insulin-like growth factor binding protein 2 (IGFBP2) protein and connective tissue growth factor (CTGF) mRNA [128], thus aiding in radiotherapy resistance.

Numerous studies have investigated the role of EV-associated miRNAs in GB progression. Among the identified EV-associated miRNAs, miR-21 stands out as a crucial oncogenic miRNA in many cancer types, including GB, through its role in stimulating tumor cell proliferation, invasion and metastasis [148,149], promoting angiogenesis [137,150,151], inhibiting apoptosis [152] and polarizing tumor-associated macrophages towards the M2 phenotype [153]. Other EV-associated miRNAs were shown to promote proliferation and invasion of GB cells (miR-19a, miR-23, miR-29a, miR-30a, miR-92b, miR-148a, miR-221, miR-222, miR-451, miR-1587, miR-5096), angiogenesis (miR-19b, miR-29a, miR-30e, miR-296) and macrophage M2 polarization (miR-1246) [23,131,137,154,155,156,157,158,159,160,161]. EV-associated miR-21 and miR-451 have been shown to inhibit c-Myc expression, resulting in increased proliferation of recipient cells and improved resistance to metabolic stress [137,162]. Additionally, miR-132 delivered by neuron-derived EVs modulates the vascular permeability in GB [163]. In contrast, other studies identified EV-associated miRNAs with anti-tumorigenic properties in GB progression, mostly derived from mesenchymal stem cells (MSCs). Thus, miR-124 [164], miR-146b [165], miR-199a [166], miR-375 [167], miR-454-3p [168] and miR-504 [169] inhibit glioma cell proliferation, invasion and migration, while miR-1 determines anti-angiogenic effects [155]. EV-associated miR-93, miR-151a, miR-193 and miR-1238 confer TMZ resistance in recipient glioma cells [27,28,29], while resistance to radiotherapy of recipient cells can be achieved through EV-mediated transfer of miR-135b [30] and miR-301a [31]. It has been shown that miR-105 [170] and miR-181c [171] are able to disrupt the blood–brain barrier and downregulate tumor suppressor genes, promoting metastasis of GB.

## 4. Therapeutic Potential of microRNA-Carrying Extracellular Vesicles against Glioblastoma

### 4.1. Extracellular Vesicles as Alternative Biomarkers in Glioblastoma Diagnosis and Monitoring

EVs can be isolated from various biological fluids [34,35,36,37,38,39,40,41] and transport specific molecules from their cells of origin, including miRNAs [23,24]. Furthermore, EVs have been shown to play an important role as mediators of intercellular communication in both physiological and pathological conditions [21,93] and are even capable of crossing the blood–brain barrier [52,53]. EVs reflect the plasma membrane, exhibiting specific surface markers and transport cargo equivalent to their cells of origin [22,115,119,120,121,122,172], which make tumor-derived EVs relatively easy to quantify, separate and characterize. Moreover, following administration of 5-ALA, GB-derived protoporphyrin IX-positive EVs can be isolated and characterized [173,174]. These properties suggest that EVs isolated from serum/plasma or CSF have great diagnostic, prognostic and disease monitoring potential and could be valuable biomarkers by themselves or through their specific cargo. Despite ample efforts to develop new diagnostic tools, minimal advances have been made and no circulating biomarkers are being used in clinical practice for diagnosis, prognosis or progression of GB [12]. Many studies have attempted to establish specific serum or CSF biomarkers for GB, but their collective findings fail to identify reliable biomarkers.

Interestingly, the number of circulating EVs has some diagnostic and prognostic value by itself. It has been shown that the number of plasma EVs was higher in GB patients compared to controls [43]. Furthermore, plasma MV number returned to baseline after surgical resection [23], was correlated with poor overall survival and earlier recurrence [175] and was indicative of tumor progression and treatment response of GB patients [176,177].

In respect to EVs’ cargo, it has been shown that GB-derived EVs transport EGFRvIII mRNA, its levels correlate with the levels found in the originating cells [23,143,178] and were indicative of poor survival of GB patients [179]. GB-derived EVs also carry mutant IDH1 transcripts [180,181], as well as specific proteins, such as EpCAM [120], TGF-β1 [182], heatshock proteins [182] and several invasion-related proteins [183]. Hallal et al. [184] identified eleven proteins exclusively found in plasma EVs isolated from GB patients, and five proteins specific to GB-derived EVs, which were correlated with their respective higher gene expression in tumors compared to normal brain tissue. Huang et al. [185] determined that polymerase I and transcript release factor (PTRF or Cavin1) levels in serum exosomes from GB patients were positively correlated with tumor grade and were decreased after surgical resection. Chandran et al. [186] determined that plasma EVs-associated syndecan-1 discriminates between low-grade and high-grade gliomas.

There are several studies published so far that investigate EV-associated miRNAs as GB biomarkers with diagnostic, prognostic and disease monitoring potential and their findings are summarized in Table 3. Six of these studies [23,46,47,48,187,188] identified increased levels of miR-21, alone or in combination with other miRNAs, in EVs isolated from serum or CSF of GB patients compared to controls (healthy subjects or non-oncologic patients). However, mir-21 is the most extensively investigated miRNA, being consistently reported to be overexpressed in different cancer types [189]. Interestingly, three of these studies [46,47,48] determined that mir-21 levels in EVs decrease after surgical resection of the tumor, suggesting its potential use as a biomarker for GB recurrence.

Lan et al. [194] measured increased levels of miR-301a in serum exosomes, which were capable of discriminating GB patients from healthy controls or other CNS malignancies, and were negatively correlated with overall survival and reflected disease progression. Zeng et al. [28] investigated miR-151a levels in paired serum and CSF exosomes from GB patients and determined that lower levels in CSF exosomes were correlated with worse prognosis and poor response to treatment. Zhong et al. [49] determined that decreased levels of miR-29b in serum exosomes distinguished GB patients from healthy controls and were correlated with poor overall survival and disease-free survival. Moreover, exosomal miR-29b levels increased after surgical resection and could be used as a disease monitoring biomarker [49]. Tabibkhooei et al. [50] identified increased levels of miR-210 in serum exosomes which were specific to GB patients, correlated with poor overall survival and fluctuated with disease progression. Li et al. [51] showed that serum exosomal miR-574-3p could be a biomarker for radiotherapy efficiency in glioma.

Other studies [48,187,188,190,191] determined miRNA signatures in EVs isolated from serum or CSF of GB patients, comprised of at least two miRNA, which were capable of differentiating GB patients from healthy controls or non-oncologic patients and had prognostic value. Additionally, Ebrahimkhani et al. [193] identified a seven exosomal miRNA signature capable of accurately diagnosing GB prior to surgical resection and discriminating GB patients from healthy controls.

However, these studies do not offer a unifying and well validated EV-associated miRNA signature, due mostly to small cohort sizes, differences in experimental design and EV isolation methods. This could suggest that these biomarkers might be dependent on the studied cohort, in terms of population characteristics and cohort size. Additionally, there is a great variation in the statistical analysis methods used and there is a lack of data regarding medication and co-morbidities for the patients included in these studies. Therefore, studies with larger, well characterized cohorts are needed in order to determine a possibly unitary EV-associated miRNA signature of GB.

### 4.2. Extracellular Vesicles as Therapeutic Tools in Glioblastoma Treatment

Beside their ability to deliver specific cargo to recipient cells [119] and to cross the blood–brain barrier [52,53], EVs have the intrinsic capacity to interact with the plasma membrane and are internalized more efficiently than synthetic lipid nanocarriers [195]. Additionally, the use of EVs as therapeutic tools for GB has additional advantages: high stability during storage and over freeze/thaw cycles, low immunogenicity and tumorigenicity, short-term effects, manufacturing scalability, possibility of using autologous cells, cargo and membrane tailoring for specificity [196,197]. However, EV use in GB therapy also has some limitations: EVs are cleared relatively fast from the circulation, decreasing their efficacy and bioavailability, and unmodified EVs have limited target specificity and loading capability [198]. In recent years, there have been attempts to mitigate these limitations by employing EV surface modifications and increasing loading capacity [198,199,200].

MiRNAs can be transported in circulation by EVs [116], thus being protected from degradation by ribonucleases. In addition, EVs can be enriched in specific miRNAs by manipulating their donor cells [54,165,201,202] or by loading EVs extracellularly using transfection, electroporation, sonication or other methods [203]. Furthermore, there are various chemical modifications that grant synthetic miRNAs the ability to avoid degradation, as well as miRNA sponges [204] or miRNA inhibitors (anti-miRNA oligonucleotides (AMOs)) specifically designed for in vivo administration [205].

**Figure 2:**
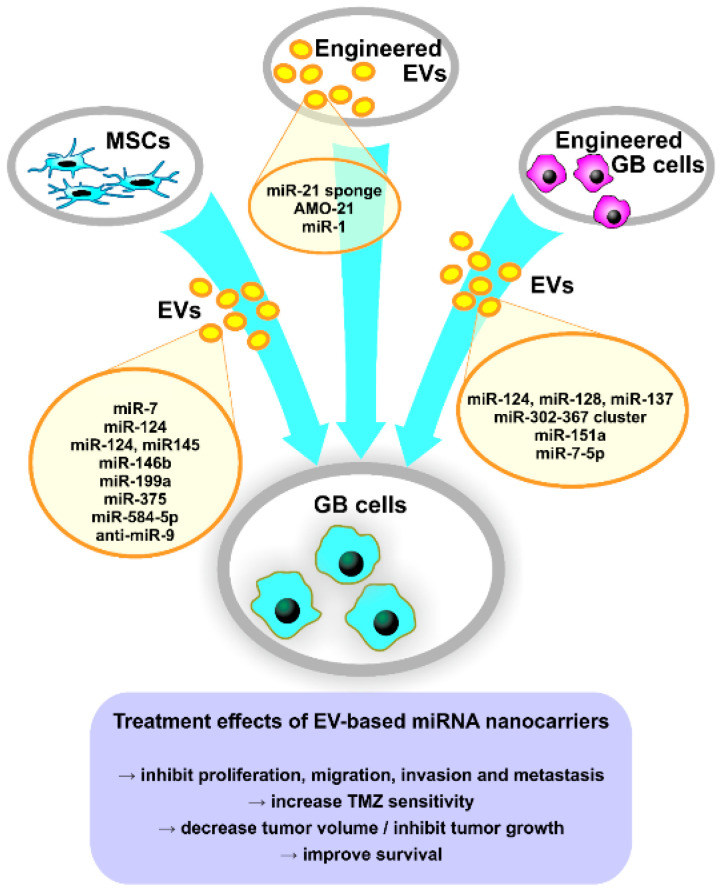
Tailoring approaches of EVs’ miRNA cargo for GB therapy. AMO-21, anti-miR-21 oligonucleotides; EVs, extracellular vesicles; GB, glioblastoma; miRNA, microRNA; MSCs, mesenchymal stem cells; TMZ, temozolomide.

Several studies investigated EVs released from MSCs of different origins as possible therapeutic carriers for endogenous or synthetic miRNAs or miRNA inhibitors, due to their low immunogenicity [196]. Thus, MSC-derived EVs carrying miR-7 [206], miR-124 [55,164,207], miR-145 [55], miR-146b [165], miR-199a [166], miR-375 [167] or miR-584-5p [208] inhibited GB cells’ proliferation, migration, invasion and metastasis, and suppressed tumor growth in animal models (Figure 2). MSCs transfected with a miR-124a-containing lentivirus vector produced miR-124a-enriched exosomes that reduced GSCs viability and clonogenicity in vitro, promoted longer survival and induced tumor regression in mice with intracranial GSC xenografts [207]. Other studies showed that miR-146b-enriched exosomes suppressed glioma growth in vitro by targeting EGFR mRNA [165], exosomal miR-375 promoted apoptosis by targeting SLC31A1 [167] and exosomal miR-584-5p inhibited metastasis by decreasing MMP2 expression [208]. Furthermore, MSC-derived EVs were able to transfer miR-124 [164,207], miR-199a [166], miR-375 [167] or miR-584-5p [208], which determined increased TMZ-sensitivity of GB cells in vitro and/or in vivo (Figure 2).

Using a different approach, Munoz et al. [56] reported that MSC-derived MVs were able to transfer synthetic anti-miR-9 to GB cells in vitro, thus decreasing P-GP expression and sensitizing GB cells to TMZ (Figure 2). Furthermore, Hamideh et al. [57] reported that engineered exosomes loaded with a miR-21 sponge are efficient in vitro on GB cell lines and reduced tumor size in a rat GB xenograft model (Figure 2). Additionally, Kim et al. [209] developed T7 transferrin receptor-binding peptide-decorated exosomes loaded with AMO against miR-21 (AMO-21) by electroporation, which had higher delivery efficiency in vitro compared to unmodified exosomes (Figure 2). Furthermore, T7-decorated exosomes delivered AMO-21 into the brain of intracranial GB rat models when administered intravenously and effectively reduced tumor size and miR-21 expression [209]. This study shows the successful targeting of GB through the blood–brain barrier by intravenously administered modified exosomes and could represent an important breakthrough in the development of new therapies based on EV-associated miRNAs.

An interesting approach is represented by manipulating GB cells to produce EVs enriched in specific miRNAs, utilizing their innate targeting toward neighboring cells, including tumor cells. Thus, Fareh et al. [54] showed that patient-derived GSCs released exosomes carrying the miR-302-367 cluster, which were internalized rapidly by neighboring cells, inhibiting their proliferation, invasion and stemness by repressing Cyclin A, Cyclin D1, E2F1 and the CXCR4 pathway. Moreover, it has been demonstrated that EV-mediated transfer of miR-124, miR-128 and miR-137 to recipient GB cells in vitro and in vivo improved survival of GB model mice when combined with chemotherapy [210], while GB-derived miR-151a-enriched exosomes induced TMZ sensitivity in resistant GB cells and in a GB xenograft mouse model [28]. Wang et al. [211] reported that exosomal miR-7-5p released from GB cells treated with verbascoside reduced EGFR expression and PI3K/Akt signaling in recipient cells, inhibiting their proliferation, migration and invasion. Furthermore, miR-7-5p-enriched exosomes reduced tumor formation and metastasis in GB nude mice compared to control mice [211].

In another study, Bronisz et al. [141] demonstrated that miR-1-enriched MVs reduced tumorigenicity and tumor microenvironment remodeling. Although not designed as treatment by the authors, miRNA replacement represents an interesting therapeutical approach for GB, using modified tumor-derived EVs as targeted Trojan horses to suppress tumor development. Another targeted approach that could be employed for GB tumors is the one designed by Ohno et al. [212], which uses exosomes modified with the GE11 peptide (an EGFR ligand) and carrying the tumor suppressor let-7 for targeting EGFR-positive breast cancer cells and reducing tumor growth in vivo. This approach could be useful in the treatment of GBs with EGFR amplification.

## 5. Conclusions and Future Directions

Despite recent advancements in diagnostic techniques, GB diagnosis is frequently done in advanced stages of the disease. There is a need for minimally invasive, easy to measure and cost-effective biomarkers for early diagnosis and therapeutic response monitoring of GB. The research data obtained in the last decades has led to the discovery of new potential biomarkers, among which miRNAs seem to be the most promising ones. Moreover, there is increasing evidence that EV-associated miRNAs may provide a more specific discrimination between studied cohorts and, therefore, could have great diagnostic and prognostic value. However, these biomarkers have not been yet introduced in clinical practice due to great differences between studies. In order to identify reliable biomarkers, larger studies with well characterized cohorts of patients need to be undertaken.

Furthermore, EV-based miRNA nanocarriers can be taken into consideration as adjuvants in GB therapy, combined with the current standard of treatment.

This review offers a comprehensive presentation on EV pathobiological significance in GB progression and resistance to therapy. Additionally, the potential of EVs and their miRNA content as diagnostic, prognostic and disease monitoring biomarkers and as nanocarriers for gene therapy is discussed.

Although the clinical use of EV-based therapeutics depends on various safety, biological and manufacturing aspects that must be clearly regulated, and the products must be tested further in vitro and in vivo, there is great promise that in the near future, EV-based miRNA nanocarriers will become part of standard clinical practice.

## Figures and Tables

**Table 1:** Molecular classifications of GB subtypes.

	Phillips et al. [105]			Verhaak et al. [5]			
Classification	Proneural	Proliferative	Mesenchymal	Proneural	Neural	Mesenchymal	Classical
Tumor type	WHO grade III or IV with or without necrosis	WHO grade IV with necrosis	WHO grade IV with necrosis	GB	GB	GB	GB
Survival prognosis	Longer	Short	Short	Longer	Short	Short	Short
Treatment efficiency	ND	ND	ND	Non-effective	Effective	Effective	Effective
Chromosome gain/loss	None	10q23.3 del (PTEN) 7p11.2 amp (EGFR)	10q23.3 del (PTEN) 7p11.2 amp (EGFR)	+7/−10 (54%) 4q12 amp (PDGFRA)	-	17q11.2 del (NF1)	+7/−10 9p21.3 del (CDKN2A)
EGFR	Normal	Amplified or normal	Amplified or normal	-	Amplified	-	Highly amplified, EGFRvIII
Mutations	-	-	-	IDH1, PDGFRA, TP53	-	NF1	EGFRvIII TP53 wildtype
Overexpressed genes	OLIG2, MAP2, DCX, ENC1, ERBB4, GAD2, DLL3, DLL1, HEY2, ASCL1	PCNA, TOP2A	PECAM, VEGF, VEGFR1, VEGFR2	PDGFRA, NKX2-2, OLIG2	-	TRADD, RELB, TNFRSF1A, CD68, PTPRC, TNF, CHI3L1, MET,	NES, NOTCH3, JAG1, LFNG, SMO, GAS1, GLI2
Markers	Markers specific to neuroblasts and immature neurons	MELK, EGFR	EGFR, VEGF, VEGFR1, VEGFR2, endothelial cell markers, CHI3L1, CD44, STAT3, vimentin	SOX genes, DCX, DLL3, ASCL1, TCF4	NEFL, GABRA1, SYT1, SLC12A5. Expression patterns similar to normal brain tissue	CD44, MERTK	EGFR/EGFRvIII, TP53 wildtype

ASCL1, Achaete-Scute Family BHLH Transcription Factor 1; amp, amplification; CDKN2A, cyclin-dependent kinase inhibitor 2A; CHI3L1, Chitinase 3-Like 1; DCX, Doublecortin; del, deletion; DLL1, Delta Like Canonical Notch Ligand 1; DLL3, Delta Like Canonical Notch Ligand 3; EGFR, epidermal growth factor receptor; EGFRvIII, epidermal growth factor receptor variant III; ENC1, Ectodermal-Neural Cortex 1; ERBB4, Erb-B2 Receptor Tyrosine Kinase 4; GABRA1, Gamma-Aminobutyric Acid Type A Receptor Subunit Alpha1; GAD2, Glutamate Decarboxylase 2; GAS1, Growth Arrest Specific 1; GB, Glioblastoma; GLI2, GLI Family Zinc Finger 2; HEY2, Hes Related Family BHLH Transcription Factor With YRPW Motif 2; IDH1, isocitrate dehydrogenase 1; JAG1, Jagged Canonical Notch Ligand 1; LFNG, Lunatic Fringe (Drosophila) Homolog; MAP2, Microtubule Associated Protein 2; MELK, Maternal Embryonic Leucine Zipper Kinase; MERTK, MET Proto-Oncogene, Receptor Tyrosine Kinase; ND, not determined; NEFL, Neurofilament Light Chain; NES, Nestin; NF1, neurofibromin 1; NKX2-2, Homeobox Protein NK-2 Homolog B; NOTCH3, Notch Receptor 3; OLIG2, Oligodendrocyte Transcription Factor 2; PCNA, Proliferating cell nuclear antigen; PDGFRA, platelet-derived growth factor receptor A; PECAM, Platelet endothelial cell adhesion molecule; PTEN, phosphatase and tensin homolog; PTPRC, Protein Tyrosine Phosphatase Receptor Type C; RELB, RELB Proto-Oncogene, NF-KB Subunit; SLC12A5, Solute Carrier Family 12 Member 5; SMO, Smoothened; SOX, SRY-Box Transcription Factor 2; STAT3, Signal Transducer and Activator Of Transcription 3; SYT1, Synaptotagmin 1; TCF4, Transcription Factor 4; TNF, tumor necrosis factor; TNFRSF1A, Tumor necrosis factor receptor superfamily member 1A; TOP2A, DNA Topoisomerase II Alpha; TP53, Tumor Protein P53; TRADD, Tumor necrosis factor receptor type 1-associated DEATH domain; VEGF, Vascular endothelial growth factor; VEGFR1, Vascular endothelial growth factor receptor 1; VEGFR2; Vascular endothelial growth factor receptor 2; WHO, World Health Organization.

**Table 2:** Main characteristics of EVs.

	Exosomes	Microvesicles	Apoptotic Bodies
Size	30–100 nm	100–1000 nm	1–5 µm
Density	1.13–1.19 g/mL	1.25–1.30 g/mL	1.16–1.28 g/mL
Origin and release mechanisms	Inward budding of endosomes to create multivesicular bodies, which fuse with the plasma membrane and release containing vesicles.	Direct outward budding (blebbing) of the plasma membrane, accompanied by cytoskeleton rearrangements and phospholipids’ relocation to the outer membrane.	Blebbing of plasma membrane during cell death, cellular debris.
Lipid membrane composition	Similar to donor cells’ plasma membrane (includes bone morphogenetic protein): lysobisphosphatidic acid, cholesterol, ceramide, sphingomyelin, phosphatidylcholine, phosphatidyl-ethanolamine, ganglioside GM3, phosphatidylinositol.	Characterized by high phosphatidylserine externalization. High levels of cholesterol, sphingomyelin, and ceramide.	Similar to parental cells’ plasma membrane (without bone morphogenetic protein): cholesterol, phosphatidylserine.
Markers	Membrane impermeable (PI negative). Alix, TSG101, CD9, CD63, CD81, CD82, CD89, flotillin, annexin, hsp70, hsp90.	Membrane impermeable (PI negative). Integrins, selectins, flotilin-2, other antigens of parental cell, phosphatidylserine.	Membrane permeable (PI positive). Histones, DNA, phosphatidylserine.
Cargo	DNA, mRNA, miRNA, lipids, specific proteins	DNA, mRNA, miRNA, lipids, specific proteins	Cellular organelles, RNA, fragmented DNA, proteins
References	[19,21,109,110,111,112,113,114,115,116]	[21,111,115,117]	[21,109,112,113,115]

ALIX, apoptosis-linked gene 2-interacting protein X; hsp70, 70 kilodalton heat shock protein; hsp90, 90 kilodalton heat shock protein; mRNA, messenger RNA; miRNA, microRNA; PI, Propidium iodide; TSG101, tumor susceptibility gene 101.

**Table 3:** EV-associated miRNAs as GB biomarkers with diagnostic, prognostic and disease monitoring potential.

miRNAs with Increased Levels	miRNAs with Decreased Levels	Source	Investigated Pathology	Diagnosis Potential	Prognostic Potential	Disease Monitoring Potential	Reference
miR-21	-	Serum MVs	GB vs. normal controls	Discriminate GB patients from normal controls.	-	-	[23]
miR-21	-	CSF EVs	GB vs. non-oncologic patients	Discriminate GB patients from non-oncologic patients.	-	Significantly reduced levels after surgical resection.	[46]
miR-320, miR-574-3p	-	Serum exosomes	GB vs. healthy controls	Discriminate GB patients from healthy controls. Not validated in second cohort.	-	-	[190]
miR-21	-	CSF exosomes	GB vs. non-oncogenic neuropathic patients	Discriminate GB patients from non-oncogenic neuropathic patients. Positive correlation with clinical grade.	-	Reduced levels after surgical resection and increased again during GB recurrence.	[47]
miR-21, miR-218, miR-193b, miR-331, miR-374a	miR-548c, miR-520f, miR-27b, miR-130b	CSF EVs	GB vs. non-oncologic patients	Discriminate GB patients from non-oncologic patients.	-	-	[187]
200–400 miRNAs unique to the disease	-	CSF EVs	GB vs. neurologically normal donors	Discriminate GB patients from neurologically normal donors.	-	-	[191]
miR-422a, miR-494-3p, miR-4443, miR-502-5p, miR-520f-3p, miR-549a	-	Serum exosomes	GB vs. normal controls	Discriminate GB patients from normal controls.	Decreased levels of miR-422a associated with poor prognosis. Increased levels of miR-502-5p associated with longer survival.	-	[192]
miR-182-5p, miR-486-5p	miR-328-3p, miR-339-5p, miR-340-5p, miR-485-3p, miR-543	Serum exosomes	GB vs. healthy controls	Discriminate GB patients from healthy controls. Accurately diagnose GB preoperatively.	-	-	[193]
miR-21, miR-222, miR-124-3p	-	Serum exosomes	GB vs. healthy controls	Discriminate GB patients from healthy controls.	Exosome-associated miR-21 levels could predict glioma grading before surgery.	Reduced levels after surgical resection.	[48]
miR-301a	-	Serum exosomes	GB vs. healthy controls/meningioma/primary diffuse large B-cell lymphoma of the CNS/pituitary adenoma	Discriminate GB patients from non-GB patients. Positive correlation with pathological grades. Negative correlation with KPS scores.	Negative correlation with overall survival.	Significantly reduced levels after surgical resection and increased again during GB recurrence.	[194]
miR-151a	-	Paired CSF and serum exosomes	GB	-	Decreased CSF exosomal miR-151a levels correlated with worse prognosis and poor response to treatment. No correlation for serum exosomal miR-151a levels. No correlation between levels in serum vs. paired CSF exosomes.	-	[28]
-	miR-29b	Serum exosomes	GB vs. anaplastic astrocytoma or healthy controls	Discriminate GB patients from healthy controls or anaplastic astrocytoma patients. Reduced levels associated with MGMT methylation status, IDH mutation status and KPS score.	Reduced levels positively correlated with short overall survival and short disease-free survival.	Increased levels after surgical resection.	[49]
miR-15b-3p, miR-21-3p, miR-155-5p, let-7a-5p	-	Serum EVs enriched by Size-Exclusion Chromatography	GB vs. healthy controls	-	miR-15b-3p, miR-21-3p, miR-328-3p—negative correlation with survival. miR-106a-5p—positive correlation with survival.	-	[188]
miR-210	-	Serum exosomes	GB vs. healthy controls	Discriminate GB patients from healthy controls.	Negative correlation with overall survival.	Reduced levels after surgical resection and increased again during GB recurrence.	[50]
miR-574-3p	-	Serum exosomes	Glioma before vs. after radiotherapy	-	-	Decreased levels after radiotherapy. Increased miR-6731-5p and miR-208b-3p levels and decreased miR-2116-3p levels after radiotherapy.	[51]

CNS, central nervous system; CSF, cerebrospinal fluid; EVs, extracellular vesicles; GB, glioblastoma; IDH, isocitrate dehydrogenase; KPS, Karnofsky Performance Status; MGMT, O6-alkylguanine DNA alkyltransferase; MVs, microvesicles.
