# Correction: Simionescu et al. The Multifaceted Role of Extracellular Vesicles in Glioblastoma: microRNA Nanocarriers for Disease Progression and Gene Therapy. *Pharmaceutics* 2021, *13*, 988

**DOI:** 10.3390/pharmaceutics16101336

**Published:** 2024-10-18

**Authors:** Natalia Simionescu, Radu Zonda, Anca Roxana Petrovici, Adriana Georgescu

**Affiliations:** 1 Center of Advanced Research in Bionanoconjugates and Biopolymers, “Petru Poni” Institute of Macromolecular Chemistry, 41A Grigore Ghica Voda Alley, 700487 Iasi, Romania; natalia.simionescu@icmpp.ro (N.S.); zonda.radu@icmpp.ro (R.Z.); petrovici.anca@icmpp.ro (A.R.P.); 2 “Prof. Dr. Nicolae Oblu” Emergency Clinical Hospital, 2 Ateneului Street, 700309 Iasi, Romania; 3 Department of Pathophysiology and Pharmacology, Institute of Cellular Biology and Pathology “Nicolae Simionescu” of the Romanian Academy, 8 B.P. Hasdeu Street, 050568 Bucharest, Romania

In the original publication [1], there was a mistake in Figure 2 as published. The cited article (ref. [208], regarding miR-34a) was retracted after the publication of the present review; therefore, Figure 2 was no longer accurate. The corrected Figure 2 appears below.


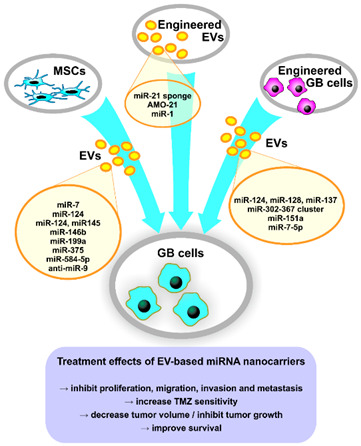


In addition to reference [208], an error was found in reference [33]. Both references were removed, citation in Section 1, together with the related text in Section 3. Extracellular Vesicles as Nano Mediators in Glioblastoma Progression, Sub-section 3.2. Extracellular Vesicles and Their Associated microRNAs as Protagonists in Glioblastoma Progression, Paragraph Number 3, and Section 4. Therapeutic Potential of microRNA-Carrying Extracellular Vesicles against Glioblastoma, Sub-section 4.2. Extracellular Vesicles as Therapeutic Tools in Glioblastoma Treatment, Paragraph Number 3.

The authors state that the scientific conclusions are unaffected. This correction was approved by the Academic Editor. The original publication has also been updated.
